# Camp stability predicts patterns of hunter–gatherer cooperation

**DOI:** 10.1098/rsos.160131

**Published:** 2016-07-13

**Authors:** Daniel Smith, Mark Dyble, James Thompson, Katie Major, Abigail E. Page, Nikhil Chaudhary, Gul Deniz Salali, Lucio Vinicius, Andrea Bamberg Migliano, Ruth Mace

**Affiliations:** 1 Department of Anthropology, University College London, 14 Taviton Street, London WC1H 0BW, UK; 2 Bristol Zoological Society, Clifton, Bristol BS8 3HA, UK

**Keywords:** cooperation, hunter–gatherers, reciprocity, mobility, demand sharing, experimental games

## Abstract

Humans regularly cooperate with non-kin, which has been theorized to require reciprocity between repeatedly interacting and trusting individuals. However, the role of repeated interactions has not previously been demonstrated in explaining real-world patterns of hunter–gatherer cooperation. Here we explore cooperation among the Agta, a population of Filipino hunter–gatherers, using data from both actual resource transfers and two experimental games across multiple camps. Patterns of cooperation vary greatly between camps and depend on socio-ecological context. Stable camps (with fewer changes in membership over time) were associated with greater reciprocal sharing, indicating that an increased likelihood of future interactions facilitates reciprocity. This is the first study reporting an association between reciprocal cooperation and hunter–gatherer band stability. Under conditions of low camp stability individuals still acquire resources from others, but do so via demand sharing (taking from others), rather than based on reciprocal considerations. Hunter–gatherer cooperation may either be characterized as reciprocity or demand sharing depending on socio-ecological conditions.

## 1. Introduction

Cooperation among organisms evolves via two routes, indirect and direct fitness benefits [1]. Indirect benefits are from kin selection [2], while direct fitness benefits occur when the act of cooperating benefits the individual as well as the recipient, which may explain cooperation among non-kin [1]. The best-known form of a direct fitness benefit is reciprocal exchange [3], a ‘tit-for-tat’ strategy [4], where individuals cooperate with others who cooperated with them previously. Reciprocity is most likely to occur when individuals have repeated interactions which foster trust and a reputation for cooperating [3]. Reciprocity avoids the free-rider problem as the long-term gains from exchanges outweigh the short-term benefits to defection [4]. While indirect fitness benefits are ubiquitous in nature [1], despite the intuitive appeal of reciprocity as a broad explanation for cooperation, it is considered predominantly applicable only to humans [5,6] (although see [7,8]). The mechanisms underlying reciprocity may, therefore, be best understood by focusing on humans.

Exploring social dynamics in the last remaining groups of present-day hunter–gatherers is essential for understanding the factors which shaped the evolution of our widespread cooperation, especially with non-kin [9–11]. Hunter–gatherer societies would not be able to survive without food-sharing which mitigates hunting stochasticity [12,13]. While reciprocal resource transfers appear to explain food-sharing behaviour in several foraging societies [8,14–16], they do not in others [17–19]. Little research has been conducted to explain the causes of this variability in forager food-sharing patterns (although see [19]). One explanation may regard differences in mobility and camp stability, which also display great variability over different foraging populations [20]. In more stable camps with fewer changes in membership it may be possible for reciprocal partnerships to build up as the same individuals interact repeatedly with one another over time, developing trust and a reputation for sharing.

High mobility poses a problem of how to receive the benefits of food transfers from others as repeated interactions are less frequent, meaning that reciprocity is less viable. One possibility may be that these societies practise a form of ‘demand sharing’ or ‘tolerated theft’ [21–23]. Under this system, individuals take resources off those who procure a larger amount of food, and the producer does not retaliate as the costs of confrontation are higher than the costs of the lost resource. Demand sharing requires an expectation that food will be shared among camp-mates, meaning that generosity is demanded, with no expectation of reciprocity [21,22]. A recent model of demand sharing found that high mobility was required for demand sharing to be viable, in order to avoid non-hunting free-riders [23]. Even under conditions of low camp stability, and without reciprocity, food-sharing can still evolve via demand sharing.

Despite seemingly disparate underlying processes, in practice, predictions made by each theory are often similar [24], as both reciprocity and demand sharing are expected when food packets are large and acquired asynchronously [25]. One means of disentangling these two mechanisms relies upon the extent to which the acquirer of the resource has control over who receives shares [16]. Reciprocity assumes that resource acquirers can select with whom to share (thus forming reciprocal exchanges), necessitating a high level of producer control over distribution. Demand sharing, meanwhile, is characterized by a lack of producer control, in that it is the recipient, not the producer, who has control over distribution.

To date, socio-ecological factors such as camp stability, which are fundamental to reciprocal exchange, have largely been overlooked in explaining patterns of hunter–gatherer cooperation. Although several experimental studies have reported an association between repeated interactions and increased cooperation in the laboratory [26,27], to the best of our knowledge no studies have reported this association using real-world data. Here we investigate cooperation among the Agta, a group of Filipino hunter–gatherers. We first explore patterns of actual food-sharing from six camps to examine whether an association between patterns of food-sharing and camp stability is present. While observational food-sharing data can elucidate the *pattern* of resource transfers, it is difficult to isolate the *process*: i.e. whether resources are given or taken. An experimental approach can be used to collect analogous data to explore these differences in the sharing process. We, therefore, also compare experimental resource transfers over 18 camps. We use semi-anonymous games in which individuals distribute resources between themselves and multiple known others. One game consists of dividing resources between ego and camp-mates, simulating giving to others (high producer control), while the other concerns taking from others in order for ego to receive resources, simulating demand sharing (low producer control). Using this methodology, it is possible to assess both how many resources individuals share with others, as well as to whom they distribute these resources, while the experimental protocol corresponds to either high producer control (giving to others) or low producer control (demand sharing). Both scenarios are required to demonstrate that food transfers occur. By including games where individuals both give and take resources from others, if individuals engage in taking behaviour, one can still infer that resources are distributed, even if individuals do not give to others.

We hypothesize that camps with greater stability will display increased reciprocity. Regarding the experimental games, in the high producer control scenario, we predict that cooperation will be higher in more stable camps as a signal of trust and expectation that others will also give to them. In the low producer control situation, we predict that taking will occur more frequently in less stable camps. We also predict that resource transfers will be reciprocal in the high producer control situation, as individuals give to others with the expectation that their generosity will be reciprocated. Transfers in the low producer control situation are not predicted to be reciprocal and depend primarily on resource quantity (take from those with more), in line with theories of tolerated theft/demand sharing.

Results support the prediction that there is increased provisioning (giving more and taking less) in stable camps and more demand sharing (taking more and giving less) in unstable camps. We also find that the profile of food-transfers differs dramatically under conditions of high versus low producer control. Reciprocity and kin-biased transfers occur under situations of high producer control, whereas the main determinants of demand sharing are resource size (taking from those with more resources) and proximity (take more from closer neighbours). These results demonstrate that individuals give to others with the expectation of reciprocity and that levels of giving increase with the probability of repeated interactions. This indicates that reciprocal cooperation is more prevalent in stable camps. These patterns of experimental resource transfers are mirrored in observations of actual food-sharing. We conclude that cooperation among hunter–gatherers is highly dependent on socio-ecological context, especially regarding the role of camp stability and the formation of long-term reciprocal sharing partnerships. Our findings provide an insight into the evolutionary basis of cooperation in human groups.

## 2. Material and methods

### 2.1. Data collection

Food-sharing data were collected in six camps (see electronic supplementary material, §1 for ethnographic background), and was conducted by asking each household at the end of each day, ‘Which households did you receive food from today?’ and ‘Which households did you give food to today?’ For verification and accuracy, focal households were asked what they had eaten that day, and who they received it from (if not procured themselves). A total of 103 days of food-sharing data were collected over these six camps.

Games were conducted with 324 Agta (mean age = 37.1, range = 16–70, males = 160) over 18 separate camps (mean number of adults = 18, range = 8–46). All adult Agta were asked if they would like to participate. Individuals who did not wish to take part were excluded as potential recipients (although very few declined). However, due to high mobility some individuals who agreed to be included to begin with were not present when the games were conducted. Thus, even though 324 Agta were included as potential recipients, games were played with 290 Agta (mean age = 37, males = 140).

For the games, participants were shown their own picture, along with a maximum of 10 other randomly selected adults from camp (individuals from camps with less than or equal to 10 other camp-mates were shown all other adults). For the Sharing Game (SG; simulating high producer control) participants were then given a number of small wooden tokens representing rice equal to the number of camp-mate's photos. Hence not every picture, including ego, could end up with rice on it, introducing a social dilemma regarding whether to share, as it would be impossible for everyone (including ego) to receive rice (e.g. with 11 pictures (10 camp-mates plus ego) 10 tokens would be given, while for 9 pictures (8 camp-mates plus ego) 8 tokens would be given). Each token represented one-eighth of a kilo of rice (125 g), described to participants as equivalent to a small cup of rice to facilitate understanding of the quantities involved. Participants then decided, token by token, whether to keep the rice for themselves, or to give to a camp-mate, and if so, to who, until there were no tokens remaining (electronic supplementary material, figure S1, upper). In the Taking Game (TG; simulating low producer control) half of camp-mate's pictures had one token placed on them, while the other half had two tokens. This was to explore the impact of resource quantity on taking behaviour (allocation was random). To acquire rice, ego had to take tokens from others and place them on ego's own picture (electronic supplementary material, figure S1, lower). The order in which the games were played was randomized. For additional discussion on designing and implementing the procedure, see the electronic supplementary material, §2.

### 2.2. Statistical analysis

Inter-household sharing was analysed using a multilevel formulation of the social relations model (SRM [28,29]) on count data using MLwiN software through R. For each camp, a matrix containing the number of days households shared food with one another was constructed (note that this is instances of food-sharing, not amount shared). A ‘shared days’ variable was entered into each analysis to control for cases where certain households were not present in camp for a day or more, so could not have received food from other households. The SRM allows for dyadic measures, such as food-sharing, to be partitioned into separate giver, receiver and relationship variance components. By assessing correlations within dyads the level of dyadic reciprocity can be estimated [29]. Kinship effects were also investigated, with relatedness between households defined as the maximum relatedness coefficient between two individuals in each household. The multilevel formulation of the SRM is suitable for small samples and count data, and removes the endogeneity bias inherent in other calculations of dyadic reciprocity [28].

Two analyses using the experimental data were performed to explore sharing behaviour among the Agta in these games; the first assessed which variables predicted the *amount* given/taken in these games, while the second explored *who* individuals gave to/took from. For the ‘amount shared’ analysis, response variables were percentage of rice kept for self and percentage of rice taken from others for the SG and TG, respectively. The main predictor variable of theoretical interest was camp stability, measured as the amount of in- and out-of-camp mobility over multiple visits to each camp (minimum two month period). This was measured by noting the residents of a camp each time it was visited and calculating the amount of change in camp composition over time, with ‘1’ meaning no change in camp composition and ‘0’ meaning complete change in camp composition (see electronic supplementary material, §3 for further details). Camp stability varied greatly between camps, from a minimum of 0.12, indicating nearly total change in camp composition, to 0.79, indicating a great degree of stability over time, with an average of 0.51 (s.d. = 0.22). Although games were conducted in 18 camps, stability measures were only available for 11 of these camps (*n* = 183, male = 90, average age = 38). As camp stability is the main variable of interest for the ‘amount shared’ analysis, only analyses for this subset are presented here. Other demographic, socio-ecological and behavioural variables were included in the analysis to explore and control for other factors influencing cooperation (electronic supplementary material, table S1). Analyses were conducted using a multilevel approach to explore behavioural variation at different hierarchical units (individuals nested within camps [30]). Univariate analyses were conducted first, with variables significantly better than a null model (with *p* < 0.1) subsequently entered in the multivariate model-averaging approach to determine the best-fitting model (see electronic supplementary material, §4 for additional details).

For the dyadic analysis of who individuals gave to/took from, response variables were coded as ‘1’ if ego gave to alter in the SG, or took from them in the TG, or ‘0’ if alter received no gifts from ego in the SG, or were not taken from by ego in the TG. Independent variables included kinship relation between ego and alter, reciprocity (whether alter gave to/took from ego), household proximity, and, for the TG, resource quantity (whether alter began the game with 1 or 2 tokens; see electronic supplementary material, §4). In order to account for differences in amounts given/taken between individuals, ego's cooperative score (% kept for self) was included as a control variable in all models. This control ensures that patterns of resource transfers were not confounded with the amount distributed, as without it reciprocity and cooperativeness may be conflated if individuals distribute widely (i.e. mistaking generalized sharing, with no expectations of reciprocation, for actual reciprocity). Although there were 2752 dyads in total (using the data from all 18 camps), as a result of the game structure, reciprocity was not possible to measure for all individuals in larger camps, resulting in 1312 dyads in analyses containing the reciprocity variable. To analyse this dyadic data a generalized estimation equation (GEE) approach was used to control for repeated data from the same individual [31]. Logistic regressions were conducted on vectors containing dyadic information regarding the relationship between ego and alter. Owing to GEE analyses not using full-likelihood estimates, quasi-likelihood information criterion estimates (QIC [32]) were employed to compare model fit. As QIC values are not comparable between models with different sample sizes, all models contained the same 1312 dyads. All possible model combinations were compared and the model with the lowest QIC value was used as the final model.

## 3. Results

### 3.1. Food-sharing results

The results of the SRM on observed food-sharing found that three of the six camps displayed significant levels of reciprocity, while reciprocity was weaker and non-significant in the other camps (table 1). Resource transfers were, therefore, much more reciprocal in three of the camps, suggesting that mechanisms other than reciprocity explain food-sharing in the other three camps, potentially including demand sharing. Kinship effects also appear stronger in the reciprocal camps (table 1). This suggests that there are differences in food-sharing practices between camps. As indicated in table 1 there appears to be an association between camp stability and reciprocity, with more stable camps increasingly likely to be reciprocal (average stability in reciprocal camps = 0.57; average stability in non-reciprocal camps = 0.28). With only six camps, it is difficult to control for camp size which also appears to be associated with sharing patterns, with larger camps being increasingly reciprocal (average number of families in reciprocal camps = 16.67; average number of families in non-reciprocal camps = 8.33). Experimental games can help disentangle these effects as more camps can be sampled.

**Table 1.** Results of the social relations model on observed food transfers in six camps. Significant effects relating to dyadic reciprocity or kinship for each camp are displayed in bold. Giver VPC (variance partition component) refers to the amount of variance in inter-household food-sharing explained by some households giving more than others. Receiver VPC refers to the amount of variance in sharing resulting from some households receiving more than others. Relationship VPC refers to the amount of variance in the model explained by the specific dyadic relationship between households, controlling for each household's specific giver and receiver variance. Note that the relationship VPC is high in the three reciprocal camps, and low in the three non-reciprocal ones, indicating that most of the variance in food-sharing in the reciprocal camps is a result of the unique relationship between households. Note also that ‘camp stability’ and ‘camp size’ values are not part of the model, but are included for illustrative purposes as comparisons between camps (*p*-value codes: ^·^<0.1, *<0.05, **<0.01, ***<0.001).

	reciprocal camps			non-reciprocal camps		
parameter	camp 66	camp 74	camp 79	camp 67	camp 67.2	camp 78
intercept	−2.86 (0.87)***	−3.87 (0.93)***	−2.2 (1.68)	−1.3 (0.47)**	−7.12 (2.24)**	−2.17 (0.91)*
shared days	0.07 (0.05)	0.05 (0.1)	0.1 (0.31)	0.23 (0.06)***	0.22 (0.13)^·^	0.25 (0.18)
kinship	4.82 (0.73)***	3.42 (1.25)**	3.57 (1.59)*	−0.06 (1.04)	6.63 (2.87)*	1.73 (0.89)*
dyadic reciprocity	0.95 (0.1)***	0.98 (0.03)***	0.8 (0.34)*	0.52 (0.33)	0.38 (0.39)	0.53 (0.34)
giver VPC	0.04	0.01	0.14	0.33	0.06	0.56
receiver VPC	0.05	0.03	0.06	0.37	0.55	0.16
relationship VPC	0.92	0.96	0.8	0.3	0.39	0.28
camp stability	0.723	0.612	0.384	0.21	0.509	0.122
camp size (no. of families)	14	23	13	6	10	9

### 3.2. Experimental games: amount given/taken

**Figure 1.**
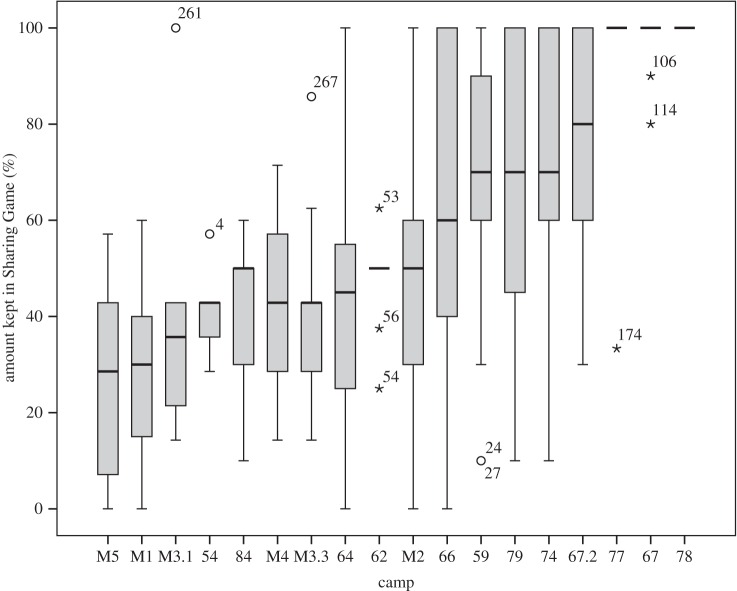
Box-plot displaying variation at the camp level in amount kept during the Sharing Game (camps = 18, *n* = 290). Sample size per camp varied between 7 and 44 (see electronic supplementary material, table S2 for full list of sample sizes and summary statistics per camp). Boxes represent inter-quartile ranges with the black lines within bars indicating the median. Lines extending above and below boxes display upper and lower quartiles, while numbered points represent outliers. Camps without boxes (62, 77, 67 and 78) had low variability in scores, so box-plots could not be produced. Camps are ordered from lowest mean amount kept (left) to highest mean amount kept (right).

**Figure 2.**
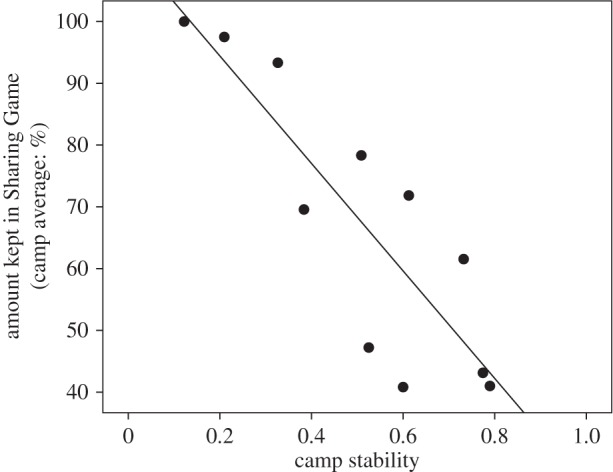
Association between camp average amount of rice kept in the Sharing Game (%) and camp stability (0 indicates no individuals remained in that camp, whereas 1 denotes camp membership was stable over multiple visits; *n* = 11).

The mean amount of rice kept by individuals in the SG over all 18 camps was 62.6% (s.d. = 30.5). This varied substantially between camps (figure 1), from a minimum camp average of keeping just 26.8% (s.d. = 20.8), to a maximum of 100% (s.d. = 0). Of these 18 camps, multilevel modelling showed that 42.9% of the variance in the null model occurred at the camp level. A Kruskal–Wallis test confirmed significant differences in amount given between camps (*H* = 121.67, d.f. = 17, *n* = 290, *p* < 0.001). Table 2 presents the results of the model-averaging procedure using the 11 camps for which camp stability measures were available (see electronic supplementary material, table S3 for results of the univariate analyses). Results indicate that a combination of camp-level and individual-level variables are associated with cooperation in the SG. At the camp level, increased camp stability and involvement in harvesting rice significantly increased cooperation by reducing the amount of rice individuals kept. For instance, a camp with no changes in composition is expected to give 67.7% points more than a camp which changed completely (figure 2), while engagement in harvesting rice increases the amount given by 20.1% points. At the individual level, each additional dependent offspring reduces cooperation and increases the amount of rice kept by 1.8% points. Stored rice was also significantly associated with sharing, as individuals without ancillary supplies kept 9.5% points more rice. Each one unit increase in affinal closeness was also associated with a 13.6% point decrease in amount given, meaning that individuals with closer affinal ties were less cooperative. None of the other variables entered in the model-averaging approach (cash labour involvement, number of primary kin in camp and average proximity to recipients) were significant predictors of cooperation in the SG.

**Table 2.** Results of the model-averaging procedure, pooled across five imputed datasets, for the Sharing Game using the sample for which camp stability data were available (*n* = 183, camps = 11). Positive parameter estimates indicate an increase in rice kept for self (%). Relative importance denotes proportion of top models used in model-averaging which contain each variable (a value of 1 means all models used for model-averaging contain this variable, while lower values indicate that less models contained this variable).

variable	level	parameter estimate	s.e.	relative importance	significance level
intercept	—	119.73	16.15	—	<0.001
camp stability	camp	−67.73	8.99	1.00	<0.001
harvesting rice (1 = no)	camp	20.15	5.73	1.00	<0.001
affinal closeness	individual	−13.59	4.48	1.00	0.003
stored rice (1 = no)	individual	9.54	3.58	1.00	0.008
no. of dependent offspring	individual	1.84	0.78	1.00	0.02
cash labour involvement	individual	−7.55	5.29	0.78	0.215
no. of primary kin in camp	individual	0.52	0.93	0.39	0.581

Similar trends emerged in the TG. Over the 18 camps an average of 65.4% (s.d. = 30) of rice was taken from others, with a minimum camp average of 35.6% (s.d. = 15.1) and maximum of 96.7% (s.d. = 12.5). Camp differences accounted for 28.1% of the variation in taking behaviour. A Kruskal–Wallis test again indicated significant between-camp differences in cooperative behaviour (*H* = 81.47, d.f. = 17, *n* = 290, *p* < 0.001). Using the 11 camps with stability data, the model-averaging results for the TG (table 3) mirrored those of the SG, with demand sharing (taking more rice) significantly associated with reduced camp stability, no engagement in harvesting rice, more dependent offspring and no rice storage. Other variables entered in to the model-averaging procedure were not significant predictors of cooperative behaviour in the TG (time spent fishing, time spent gathering or affinal closeness). This correspondence in predictor variables indicates that both games are measuring similar aspects of cooperation, as also evidenced from the significant correlation between SG and TG scores (*r* = 0.59, *n* = 290, *p* < 0.001). Thus, individuals who gave lots to others in the SG took little in the TG. Indices of genealogical kin presence, camp size or cash labour involvement had no significant association with cooperation in either game (tables 2 and 3; electronic supplementary material, table S3).

**Table 3.** Results of the model-averaging procedure, pooled across five imputed datasets, for the Taking Game using the sample for which camp stability data were available (*n* = 183, camps = 11). Positive parameter estimates indicate an increase in rice taken from others (%). Relative importance denotes proportion of top models used in model-averaging which contain each variable (a value of 1 means all models used for model-averaging contain this variable, while lower values indicate that less models contained this variable).

variable	level	parameter estimate	s.e.	relative importance	significance level
intercept	—	85.02	15.15	—	<0.001
camp stability	camp	−66.08	9.94	1.00	<0.001
no. of dependent offspring	individual	2.87	0.8	1.00	<0.001
harvesting rice (1 = no)	camp	17.54	5.78	1.00	0.003
stored rice (1 = no)	individual	10.41	3.71	1.00	0.006
gathering involvement	individual	−11.5	6.47	0.87	0.127
affinal closeness	individual	−2.36	4.11	0.38	0.573
fishing involvement	individual	1.87	3.84	0.33	0.65

Mann–Whitney *U* tests reported that the order in which games were conducted had no bearing on either SG (*U* = 4430.5, *p* = 0.47) or TG scores (*U* = 3716, *p* = 0.18), nor did familiarity with the researchers from fieldwork the previous year (SG: *U* = 10 398.5, *p* = 0.85; TG: *U* = 9303.5, *p* = 0.16). Game order and familiarity did not, therefore, confound these results. Although distance to town was associated with cooperative behaviour in the univariate analysis (electronic supplementary material, table S3), it was not included here due to significant collinearity with camp stability (*r* = −0.77, *n* = 11, *p* = 0.006). Camp stability was included in these analyses due to its greater theoretical relevance for reciprocity—and cooperation more generally—and because camp stability in the univariate analyses gave a better model fit than distance to town for the TG (stability AIC = 1718.9, distance AIC = 1721.9), while they were equivalent for the SG (stability AIC = 1711.9, distance AIC = 1711.1; see below for further discussion on the association between camp stability and distance to town). None of the other predictor variables in either the SG or TG analysis displayed heightened colinearity, suggesting that, for example, stored rice was not correlated with camp stability (electronic supplementary material, table S4).

### 3.3. Experimental games: identity of recipient

Model selection procedures are displayed in the electronic supplementary material, table S5 (for the SG) and electronic supplementary material, table S6 (for the TG). These indicate that the best-fitting model regarding who individuals gave to in the SG contained kinship and reciprocity variables, while taking behaviour in the TG was primarily a result of resource quantity and proximity effects (table 4). Reciprocity effects were found in the SG, with individuals 50% more likely to give to those who also gave to them. No effects of reciprocity were reported for the TG. Although kin effects appear large across all kin categories in the SG (electronic supplementary material, figure S2), once other terms are added to the model the role of affinal kin is greatly diminished. In the final model, a preference towards giving to primary and distant kin remain, with primary kin 3.8 times more likely to receive resources than unrelated individuals, while the corresponding value for distant kin is 1.8. Kin effects in the TG were much less pronounced compared with the SG (electronic supplementary material, figure S2), and the best-fitting model for the TG did not include any kinship effects. In the TG, the effect of starting resource quantity was large, with individuals who began the game with two resources taken from 10.8 times more often than individuals with only one resource. Although relatively small, proximity effects were found in the TG, with each increasing unit in closeness associated with a 16% increase in the likelihood of being taken from.

**Table 4.** Results of the best-fitting GEE models for the Sharing Game and Taking Game (*n* = 290, dyads = 1312). Higher log-odds estimates in the Sharing Game indicate an increased likelihood of ego giving to an alter of that category, while higher coefficients for the Taking Game are associated with an increased propensity for ego to take from alter. These models contain controls for the amount each individual gave/took (not displayed here). Dashes indicate that the variable was not included in the final model. 95% CIs are displayed in brackets.

variable	Sharing Game log-odds coefficient	Taking Game log-odds coefficient
intercept	2.43 (2; –2.87)	−3.55 (−4.23; −2.87)
reciprocity (1 = reciprocal giving/taking)	0.39 (0.1; 0.68)	—
primary kin (ref. unrelated individuals)	1.33 (0.76; 1.91)	—
distant kin (ref. unrelated individuals)	0.59 (0.04; 1.14)	—
primary kin's spouse/spouse's primary kin (ref. unrelated individuals)	0.31 (−0.25; 0.87)	—
spouse's distant kin/other affines (ref. unrelated individuals)	0.08 (−0.32; 0.48)	—
spouse (ref. unrelated individuals)	−0.16 (−0.83; 0.52)	—
resource quantity (1 = high)	n.a.	2.38 (1.87; 2.89)
proximity	—	−0.15 (−0.3; 0.00)

### 3.4. Correspondence between food-sharing and experimental data

The actual food-sharing results mirror game behaviour, as individuals in the three reciprocal camps kept less for themselves in the SG compared with those from the three non-reciprocal camps (reciprocal camps: mean = 68.2%, s.e. = 3.2, *n* = 87; non-reciprocal camps: mean = 90.5%, s.e. = 2.6, *n* = 44: *t* = −5.38, *p* < 0.001) and took less from others in the TG (reciprocal camps: mean = 66.5%, s.e. = 3.1, *n* = 87; non-reciprocal camps: mean = 87.9%, s.e. = 3.4, *n* = 44: *t* = −4.64, *p* < 0.001). Demand sharing in the games—taking more resources from others—was, therefore, more prevalent in camps which engaged less in real-life reciprocity, while camps displaying reciprocal resource transfers engaged more in giving resources to others in the games. The stronger effect of kinship in the reciprocal camps is also consistent with the findings of the dyadic game analysis that kinship effects are more pronounced under conditions of giving rather than demand sharing. Thus, behaviour in these experimental games appears to reflect patterns of actual food-sharing.

## 4. Discussion

These results suggest that camps with increased stability displayed increased reciprocal cooperation. Three lines of evidence have been offered in support of this proposition. Firstly, more stable camps appeared to display increased reciprocity in actual food-sharing analyses. Secondly, individuals from more stable camps were increasingly likely to give resources to others in the SG and less likely to take in the TG. This is consistent with theoretical accounts of reciprocity [3], in which repeated interactions promote cooperation, as trust and a reputation for sharing must be displayed for reciprocity to occur [26,27]. Thirdly, we demonstrate that giving behaviour in the SG is associated with a greater likelihood of reciprocity, suggesting that giving behaviour is predicated on expectations of reciprocity. Conversely, instability in camp composition was found to mitigate reciprocal cooperation, with camps possessing greater turnover in membership associated with reduced reciprocity in actual food-sharing analyses. We acknowledge that a lack of reciprocity in the real-world food-sharing analysis does not necessarily mean that a demand sharing system of distribution is present, although it is consistent with the results. To explore demand sharing in greater detail the *amount* shared, rather than just instances of sharing (as employed here), would be required [18,33,34]. However, as the level of taking in the TG increased in less stable camps, we infer that the lack of reciprocity in actual food-sharing reflects a greater emphasis on demand sharing in these camps. These differences were also reflected in the dyadic analysis for the TG, as reciprocal considerations were of no importance regarding whom to take from, but rather resource quantity and proximity predicted taking behaviour, consistent with theories of tolerated theft/demand sharing [22,25].

Hunter–gatherer food-sharing thus appears to fall on a continuum between demand sharing and provisioning based on reciprocity (and kinship), as further evidenced by the correlation between amount kept in the SG and amount taken in the TG. The level of sharing versus taking depends on socio-ecological context, specifically regarding stability of sharing partners. As an ethnographic example of this flexibility, reservation-living Ache with reduced mobility share meat according to reciprocal partnerships [15,35], indicating high producer control, while highly mobile forest-dwelling Ache did not display this reciprocal pattern as meat was distributed widely between all camp-mates without recourse to reciprocity [19]. This latter pattern is indicative of demand sharing and low producer control and is consistent with the analyses presented here. If individuals are obliged to share food (demand sharing), high between-camp mobility may be necessary to avoid non-hunting free-riders [23], while in situations where stability is higher, reciprocal exchanges are required to combat non-sharers [3]. This context-dependent flexibility affords protection against free-riders under a range of socio-ecological conditions, while still enabling food transfers.

Understanding the reasons for variation in camp stability is also necessary. One reason may be resource variability, as foragers are theorized to be more mobile when resources are scattered and returns more variable [36]. Analysis of foraging return rates suggests that this is unlikely with the Agta as there is no association between foraging return rates and camp stability (see electronic supplementary material, §5). Given the significant association between stability and distance from town it is plausible that integration with recently introduced institutions, such as evangelical church groups, schools, healthcare availability and labour opportunities are influencing Agta mobility patterns, as these effects are more pronounced nearer town. This suggests that the association between camp stability and distance to town is a single phenomenon, rather than separate effects. Similar patterns of exogenous influences on forager settlement patterns have been reported in other populations, such as governmental influences among the San [37] or interactions with Bantu farmer villages among central African Pygmies [38]. While understanding the causes of variation in camp stability is important, the main topic of interest here is the *consequences* of this variation. We leave it to future studies to further explore the causes of residential mobility, but nonetheless predict that when camp stability is high, regardless of the cause, greater levels of reciprocal cooperation are expected.

It could also be argued that this variation in cooperation is a result of ‘market integration’ (MI)—being exposed to market norms which encourage fairness—rather than camp stability. Although some cross-cultural studies have reported an association between cooperation in games and MI [39,40], other within-society analyses have reported no relationship [41,42]. Here, we control for various correlates of MI, including cash labour involvement, which has no effect on cooperation. A further correlate of MI is amount of food acquired by trade [40], yet this is relatively constant among the Agta as all trade foraged goods for rice with their agricultural neighbours, regardless of distance from town. Market integration is also believed to influence ephemeral interactions with strangers, rather than norms regarding resource transfers [43], so is unlikely to alter local food-sharing practices which are explored here (see also [44]). Thus, controlling for these effects, MI does not appear to impose exogamous sharing norms on the Agta; rather, increased involvement with outside institutions encourages sedentism, leading to endogenous changes in cooperation and food-sharing practices.

An important issue concerns whether these patterns of experimental resource transfers using rice are representative of wider hunter–gatherer food-sharing in other contexts, such as foraged resources. The Agta have a long history of trade with their agricultural neighbours over approximately the past 2000 years [45,46] and it has been argued that hunter–gatherers in rainforest environments, such as the Agta, may not have been able to survive without obtaining carbohydrates from farmers [47]. Although this trade has probably intensified in recent years with increasing population growth and expansion of farming populations, this suggests that the sharing of non-foraged resources among the Agta has a strong historical precedent and may be equally as salient as sharing foraged resources. The correspondence between experimental game behaviour and real-world food-sharing indicates that rice is not distributed differently relative to other resources, meaning that the sharing of rice in these games may be a valid proxy for food-sharing more generally. Similarly, among the horticultural Sursurunga in Papua New Guinea, distributions of money (a recently introduced resource) and betel nut (a traditional and widely shared resource) were equivalent in both Dictator and Ultimatum Games [48]. In other societies and with other goods, however, resources may be distributed differently from one another. Among Kenyan Samburu pastoralists, money in a Dictator Game was shared differently depending on whether contextual cues of meat-sharing were present or not [49]. This topic requires further research to determine whether and under what conditions there exists variation in sharing patterns between different resources in experimental contexts. Nevertheless, among the Agta specifically the experimental distribution of rice does appear to reflect wider food-sharing behaviour.

Measures of resource availability and need are also associated with cooperativeness. Camps engaged in harvesting rice and households with an existing supply of rice have increased resource availability, reducing the cost of cooperation. Analogously, affluent neighbourhoods in UK cities were more cooperative than more deprived neighbourhoods [50,51]. Increased local resource competition can reduce cooperation [52] and this may increase demand sharing in hunter–gatherers.

Our finding that individuals with fewer dependent offspring were more cooperative is comparable with results found among Tsimane forager-horticulturalists, albeit at a coarser scale, in which unmarried individuals gave more in the Dictator Game than married individuals [53]. These findings correspond well with recent reports of forager individuals unencumbered with dependent offspring and at a net-surplus provisioning those with many children who would otherwise be in deficit [12,54]. This is consistent with theoretical accounts of reciprocity, which indicate that reciprocity is more likely to occur when individuals are in surplus and the recipient in deficit, meaning that the benefit of giving to the recipient is higher than the cost to the donor [3,55]. Thus, individual-level cooperation appears to be in part needs-based.

Other theoretically relevant variables were not associated with cooperation. Most notable was that the presence of kin did not increase cooperation. In the SG individuals with *less* affinal kinship ties were more cooperative, potentially because of increased resource competition between closer affines. This is comparable to the effect of low dispersal increasing competition, rather than facilitating cooperation, between relatives [56,57]. Similar reasoning may also explain why cooperativeness was not influenced by levels of genetic kin in camp. Although consanguineal kin presence did not influence the *amount* given in the experimental games, Agta preferentially gave to kin *when* they distributed resources, both in the SG (see also [58,59]) and in patterns of actual food-sharing. As predicted by kin selection [16], and contrary to some previous studies [15], in both of these analyses transfers to kin were largely independent from reciprocal considerations. By contrast, resource transfers in the TG were predominantly dependent upon resource quantity and proximity, rather than kinship and reciprocity, supporting predictions of tolerated theft/demand sharing [21,22,25]. Actual food-sharing patterns showed that, although kin effects were present in most camps, they were stronger in camps which displayed heightened reciprocity. This suggests that assortative food-sharing—in terms of kin-biased and reciprocal transfers—was more pronounced in camps which possessed greater producer control, as inferred from these camps giving more in the SG (and taking less in the TG). Our findings suggest that both direct and indirect fitness benefits may be important in determining patterns of hunter–gatherer cooperation, particularly when producer control is high, although when producer control is low indirect benefits appear of lower importance.

Also unexpected was a lack of camp size effect despite a theoretically increased risk of free-riders [60]. Although larger camps appeared to rely upon reciprocity more than smaller camps from the food-sharing data, larger camps were equally as cooperative as smaller camps in the games. Of the camps tested here, group sizes (minimum = 25, maximum = 119, average = 51.9) fall within the range of other hunter–gatherers (minimum = 13, maximum = 250, average = 37.5; [61]), albeit with a slightly higher mean, suggesting that group size may not be a limiting factor to cooperation in hunter–gatherers within the observed range. Rather, ecological and demographic factors based on group stability and needs-based provisioning played a key role in the evolution of cooperation, in what was the dominant human lifestyle up to approximately 12 000 years ago.

In addition to being the first study observing an effect of camp stability on reciprocal cooperation, this is also the first study reporting a quantified correspondence between experimental indices of cooperation and real-world behaviour in a small-scale population. This association is presumed, and often inferred qualitatively, but rarely tested in a quantifiable manner. Previous studies using traditional, anonymous, experimental economic games, such as the Dictator Game and the Ultimatum Game, have found little correspondence between experimental and real-life cooperation behaviour in small-scale societies [62–64] (although this association has been reported in large industrial societies [65]). We demonstrate here that by adapting experimental games and making them relevant to the socio-ecological context (non-anonymous distribution of resources, as found in hunter–gatherer societies), it is possible to implement an ecologically valid methodology which furthers our understanding of human cooperative evolution.

## Supplementary Material

File 1: Supplementary Material Section 1: Study Population Section 2: Game Rationale and Data Collection Section 3: Camp Stability Measure Section 4: Statistical Analyses Section 5: Camp Stability and Foraging Return Rates Tables S1–S7 Figures S1 & S2

## Data accessibility

The data supporting this article are available in the Dryad Digital Repository (http://dx.doi.org/10.5061/dryad.pf67c).
