# Supplementary material for: Camp stability predicts patterns of hunter–gatherer cooperation
Source: R Soc Open Sci. 2016 Jul 13;3(7):160131. doi: 10.1098/rsos.160131 (PMC4968462; doi:10.1098/rsos.160131)
Supplement: File 1: Supplementary Material Section 1: Study Population Section 2: Game Rationale and Data Collection Section 3: Camp Stability Measure Section 4: Statistical Analyses Section 5: Camp Stability and Foraging Return Rates Tables S1–S7 Figures S1 & S2 [file rsos160131supp1.docx]

Supplementary Material for “Camp Stability Predicts Patterns of Hunter-Gatherer Cooperation”

*Section 1: Study Population*

The Agta are an indigenous Filipino population, believed to be descendents of the first colonisers of the Philippines over 35,000 years ago [1], and are distinguishable from their non-Agta neighbours by their short ‘pygmy’ physique, dark skin, tight curly hair, and predominantly foraging mode of subsistence. The Agta in the study population are from the Northern Sierra Madre Natural Park in Isabela Province, north-east Luzon, a remote area of protected forest land accessible only by plane, boat, or a three-day hike. Specifically, the study focuses on two sub-populations, the Palanan Agta, who number ~1,000 individuals, and the Maconacon Agta, who number ~250 individuals. Both sub-populations live on or near river-banks or coastal areas, and predominantly engage in foraging activities, particularly fishing, but also hunting, collecting honey, and gathering wild plants, which they either consume or trade for rice with the local agricultural non-Agta population. Depending on availability, some Agta also participate in wage labour (often clearing land for non-Agta farmers) or assist in rice harvesting, where they often receive a share of the harvest. The Agta engage in a flexible mixed subsistence economy, meaning that individuals or camps involved in harvesting rice (or wage labour) still forage alongside this agricultural/labour work, while often returning to a predominantly foraging mode of subsistence post-harvest (or post-labour). Camp sizes vary between solitary dwellings (7 individuals) and large camps of up to 26 houses (156 individuals), with an average of 7 houses (49 individuals). Mobility rates vary, with Agta closer to the main town in the area often being less mobile than those further from town (discussed in main text). Data collection spanned two fieldwork seasons, between April-June 2013 (during which demographic data were collected) and February-October 2014 (during which food-sharing, experimental game, and additional demographic data were collected).

*Section 2: Game Rationale*

After preliminary trials with different resources, it was decided that rice would be used as the game resource, as it is highly sought-after by the Agta, and therefore carries enough value to cause a dilemma when deciding to share or not. Initial trials with other goods, such as honey sticks, were perceived to have little value (and were freely distributed to children). After discussing with the Agta which resources were most valued, rice was the unanimous choice. The Agta do not grow their own rice (although they may harvest it for neighbouring agricultural populations), and although it is a non-foraged commodity introduced by non-Agta agricultural populations it is one of the Agta’s primary sources of calories (when available) and is highly-valued. The vast majority of meals are consumed with rice (and in some cases consist solely of rice), regardless of distance from town or involvement in harvesting rice. As shown below (table S4), there is no colinearity between possessing stored rice and other variables, such as camp stability or involvement in harvesting rice, indicating that rice is consumed by all Agta in this population. Differences in familiarity or cultural salience with rice are therefore likely to be minimal. One-eighth of a kilo of rice (125g) was used as this is approximately the size of a portion of rice for one adult. Filipino’s, at least in the area we worked, often weigh food in terms of fractions of a kilo (one-half, one-quarter, one-eighth, etc.), meaning that other terms of measurement, such as grams, may more likely be misunderstood.

Two non-anonymous games, known as the ‘Sharing Game’ (SG) and the ‘Taking Game’ (TG), were designed to be as simple and intuitive to grasp as possible, to minimise the need for complex explanations which may lead to frustration, boredom, and an increased risk of misunderstandings. The SG is structurally similar to the ‘Gift Game’ played with the Hadza [2], BaYaka [3] and Saami [4], where participants were given resources (e.g., sticks of honey), and had to decide who to give it to. Although the SG is structurally alike, it possesses the added rule that participants could either keep a share for themselves if they wished, or give it to a camp-mate of their choosing. Although the Gift Game allows the choice of giving to multiple individuals, it does not measure levels of cooperation as there is no option for keeping gifts for one’s self, and is therefore not a social dilemma [5], as there is no conflict between individual and group interests. On the other hand, although traditional economic games, such as the Ultimatum Game, Dictator Game, and Public Goods Game [6], are social dilemmas, they include only anonymous partners, and therefore ignore the role that differences in relationship have on cooperation [7] and cannot be used to explore who individuals preferentially share resources with. The TG is similar to the SG, but in order to get resources for self ego has to take them from others (see also [7]). Thus, the SG examines giving behaviour and resource transfers under high producer control, while the TG examines taking behaviour and resource transfers under low producer control.

*Data Collection*

Only camps with 8 or more adult members present were included in the games in order for the statistical analyses comparing groups to have a sufficient sample size to permit meaningful results. Due to the majority of Agta not knowing their exact ages, adults were defined as either married or divorced individuals, or those believed to be over the age of ~16. Approximately ten days was spent at each of the 11 Palanan camps used in the ‘amount shared’ analysis, during which time demographic, subsistence, and other measures used as independent variables in the analysis (see table S3 below) were recorded collectively by the Hunter-Gatherer Resilience Team as part of a larger research project. The other camps (one from Palanan and six from Maconacon) were only visited by the main author, where it was not possible to collect subsistence data or visit repeatedly to collect indices of camp stability (all other measures were collected for these camps, however). The games were only played on the last few days, in order to maximise familiarity with the researchers and facilitate trust, but also to minimise the potential for collusion between camp-mates. We do not believe that this occurred, as there were no sudden shifts in game behaviour over time. Prior to playing the game in each camp, photographs of all players were taken and Polaroids printed.

Although it may have been preferable to include all individuals from larger camps, rather than a sample of 10 individuals, this was chosen for practical and comparative reasons. Firstly, including all camp-mates from larger camps would have been unfeasible as the amount of rice needed would increase exponentially with camp size. Secondly, although it would have been possible to limit the amount of rice by including all individuals in a large camp and only using 10 tokens, this would make comparisons between larger and smaller camps difficult as otherwise the rule of equal tokens to potential recipients would be violated. The TG would also not work if the amount of rice was limited in larger camps. Thus, in camp with 12 or more members, 10 randomly-selected camp-mates (in addition to ego) were chosen.

For the games, individuals were taken somewhere private, away from other camp-mates. Only the participant, experimenter, and translator were aware of an individual’s decisions. Individuals were briefed on the games in their local language, and ensured that all decisions would remain secret from other camp-mates, told that there were no correct answers, and that they, and whoever they gave rice to, would be given it before the researchers left camp. If there were no questions, the games were then played. Once finished with both games, participants were thanked and asked politely not to tell anyone else about how they played the game. In total, the procedure took about 10-15 minutes per participant. Prior to leaving camp, the amount of rice earned by each participant was given to them, along with remuneration for their time and assistance in other aspects of the project conducted simultaneously.

*Section 3: Camp Stability Measure*

For each visit to a camp, individuals were scored as ‘1’ if they were present, or as ‘0’ if absent (individuals present at later visits, but not prior visits, were retrospectively given a ‘0’ for the preceding visits). This census was conducted in a cross-sectional manner upon entering a camp, meaning that subsequent in- and out-of-camp mobility during our visit would not be counted (for instance, an individual who entered the camp in the morning of day 3 and left in the afternoon of day 3 would not be included in calculations of camp stability). Each cross-sectional census occurred at least two months apart. For camps where only two visits were made, the camp stability score was simply the average of those who had moved either in or out of camp (‘0’) and those who had not moved (‘1’). For example, in a camp originally consisting of 10 individuals at time 1, if 5 had moved before our second visit, 5 individuals would have scored a ‘1’ (no change in camp) and 5 would have scored a ‘0’ (moved camp). This makes for a summed score of 5 ((5*1)+(5*0)), which, when divided by the number of camp-mates (10) gives an average of 0.5. Thus, the stability score for this camp would be 0.5. As another example, given a camp again consisting of 10 individuals on the first visit, if 5 more individuals were to join the camp and none of the original members left, 10 people would be given a score of ‘1’, and 5 would be given a ‘0’. The summed score is therefore 10 ((10*1)+(5*0)), and the average is 0.667 (the summed score (10), divided by the total number of camp-mates (15)). As a final example of a two-visit camp stability score, consider both in and out-of-camp mobility simultaneously. Again, start with a camp of 10 individuals at first visit, have 5 people leave and 5 new individuals join. The 5 people remaining across both visits would be given a ‘1’, while both the 5 who left and the 5 who joined would be given a ‘0’, so the summed score would be 5 ((5*1)+(10*0)), which, when divided by the total number of camp-mates (15), would give a stability score of 0.333.

Things become slightly less straightforward when considering three (or more) visits to a specific camp, although the principle remains the same. We can derive an estimate of individual mobility if we use the formula: 1-(ratio of 0’s to 1’s). For example, say an individual had the pattern ‘present-absent-present’ (‘1-0-1’), the ratio of 0’s to 1’s is therefore 1:2, or 0.5, and 1-0.5=0.5, so they would receive an individual mobility score of ‘0.5’. Present-present-absent (‘1-1-0’) or absent-present-present (‘0-1-1’) would also receive a score of ‘0.5’ via the same method. Given four visits, a string of absent-present-present-present (‘0-1-1-1’) would produce a ratio of 1:3, or 0.333, which, when subtracted from 1, gives a score of ‘0.667’. Meanwhile, a string of present-absent-present-absent (‘0-1-0-1’) gives a ratio of 2:2, or 1, giving a score of ‘0’ when subtracted from 1. However, when the number of 0’s is larger than the amount of 1’s, the formula does not work, but as these individuals are more often away from camp than present, their mobility is therefore high, so can be assigned a value of ‘0’. Thus, if an individual had a string of ‘0-1-0’ (Absent-Present-Absent), they would receive a score of ‘0’. As with the two-visit example in the previous paragraph, to calculate camp stability, simply sum up the individual mobility scores of each individual who lived in that camp at one time, and average this by the total number of individuals who had lived there.

Five of the camps were visited three times (once in 2013 and twice in 2014), two camps were visited twice in different years (once in 2013 and once in 2014), three of the camps were visited twice in the same year (2014), and one camp was visited four times (once in 2013 and three times in 2014). The minimum length of time between two visits was ~2 months. Although the number of visits and length between visits varied across camps (table S7), this is unlikely to bias the results, as it may be expected that changes from one year to the next would be greater than changes over only a couple of months, yet this is not the pattern observed. The three camps visited in the same year showed greater instability than camps visited in different years (same year average=0.28, *n*=3; different year average=0.59, *n*=8), a pattern likely found because the camps only visited in the same year were farther from the main town. Although seasonality can influence hunter-gatherer mobility patterns [8], and the Agta are generally less mobile during the rainy season from October to February [9], the present research was conducted during the dry season and there were no systematic biases between the months in which stable or unstable camps were visited (table S7). Differential mobility between camps cannot therefore be attributed to seasonality effects, such as climate or variation in wage labour opportunities (e.g., rice harvests). Furthermore, each dwelling was ranked on eight criteria (hut/house style (compared to lean-to), wall presence, planked walls, planked floor, metal roof, size, own dwelling (compared to shared), and owner of multiple dwellings), with a score of ‘0’ on each item representing absence, and ‘1’ signifying presence. A total score of ‘8’ therefore represented a nuclear family owning two houses, the main one of which is large house with planked walls, planked floor, and a metal roof, while ‘0’ represented a small lean-to consisting of a sheet of dried leaves propped up by a stick. As would be predicted, individuals living in more basic and temporary dwellings were significantly more likely to reside in camps with increased instability, while individuals living in larger, more permanent, houses were more likely to live in stable camps, as indicated from a linear regression between house type and camp stability (*b*=4.04, SE=0.57, *p*<0.001, *n*=183). Thus, individuals living in temporary houses lived in less stable camps, as would be predicted if individual mobility was higher in these camps, providing further evidence that the camp stability measure is a real effect, and not a methodological or sampling artefact.

*Section 4: Statistical Analyses*

For the ‘amount shared’ analyses, demographic, socioecological, and behavioural variables – including age, sex, number of dependent offspring, three indices of resource availability (involvement in harvesting rice for agricultural neighbours, whether the house had any stored rice, and money possessed by household), camp size, kin effects (average relatedness to sample, number of primary kin in camp, and affinal relatedness to sample), distance to town/trading post, average proximity to sample, presence of church in camp, location of camp (coastal or inland), household wealth, height-weight ratio, education level, average primary kin score for each game, and engagement in various subsistence tasks – were also recorded to investigate and control for these effects (see table S1 for additional details and definitions).

Analyses were conducted using the statistical software *R* [10]*.* A multilevel approach using the package *lme4* [11] was utilised to explore behavioural variation at different hierarchical units (individuals nested within camps; Kreft & de Leeuw 1998). Univariate analyses were conducted first, with variables significantly better than a null model (with a *p*-value of <0.1) subsequently entered in the model-averaging approach. Model-averaging analyses were then conducted using the package *MuMIn* [13]. Model-averaging utilises an information-theoretic approach in which various models are weighted according to how well they fit the data. The best-fitting models (models within 2 AICc values of the top model) are then averaged, meaning that parameter estimates are obtained and weighted across different models. This makes this approach superior to stepwise methods which do not consider model uncertainty [14]. A ‘zero method’ of model averaging was utilised, in which parameters absent in a subset of the top models are substituted with a value of ‘0’. The ‘zero method’ of model-averaging utilised here is a more stringent method than the alternative ‘conditional method’ which only averages parameters over the models they appear in while ignoring these parameters in top models in which they are absent. The ‘zero method’ therefore reduces the effects sizes of weakly predictive variables, meaning that significant variables are those possessing the strongest association with the response variable, reducing the possibility of type I errors [13,14].

As a result of missing data a multiple imputation procedure was employed to estimate missing values, creating five data sets. Although the amount of missing data was small (184 cases out of 7,830, or 2.3% of all predictor variables), missing data renders comparisons between models impossible using the information theoretic (IT) approach [15] employed here. A multiple imputation procedure was utilised in which missing data were imputed and *M* datasets created. Multiple imputation was carried out using the *R* package *Amelia* [16], which uses an expectation maximisation algorithm to approximate maximum likelihood estimates for the missing values. Using this method five datasets were created with no missing values, as it has been demonstrated that between 3-10 datasets is generally adequate to approximate variation in missing values [17]. This multiple imputation method is less prone to error than other data imputation methods, such as removing all cases with missing values, using the mean for missing values, or single imputation, and has been shown to recover accurate parameter estimates and IT-related measures using a real biological dataset [18]. Once analyses were conducted on each of the imputed datasets, parameter estimates, standard errors, and other indices were then pooled across each of the five datasets.

Turning now to the coding of variables in the dyadic analyses of these games, kin relationships were defined as: primary kin (PK), with a relatedness coefficient of *r*=0.5 to ego; distant kin (DK), with a relatedness coefficient between *r*=0.25 to *r*=0.03 (second cousins) to ego; spouse; spouse’s primary kin/primary kin’s spouse (SPK/PKS); spouse’s distant kin/other affines (SDK/OA), which includes distant kin of spouse or other affinal relationships up to 5 steps away from ego (e.g., spouse’s brother’s wife’s mother (4 steps away)); and non-relatives (NR), which includes everyone else without a kinship link to ego (see [19] for further details). Proximity was coded from one to four, reflecting increasing household distance between ego and alter, with categories of; living in the same house as ego (1), living next to ego (2), having a house between ego’s and alter’s (3), and living further away (4). All other details regarding statistical procedures used for these dyadic analyses are described in the main text.

*Section 5: Camp Stability and Foraging Return Rates*

Foraging data was collected over 8 camps and 457 foraging trips, which included all foraged food, such as fishing, hunting, marine resources (such as collecting shells, sea-snails, shrimps, etc., but excluding fishing), gathering wild food, and honey collecting. Variation in return rates for each camp were obtained by calculating the coefficient of variance (CV), which is the standard deviation in calories acquired per person per hour of foraging divided by the mean number of calories per person per hour of foraging. A high CV indicates greater variability in foraging return rates. Despite the theorised association between mobility and foraging return rates [20], among the Agta we find little association between the amount of variability in foraging return rates and camp stability, as there was no correlation between the two (*r*=0.21, *n*=8, *p*=0.62). Although the sample size is small, similar correlations between camp stability and distance to town indicate a strong association (*r*=-0.768, *n*=11, *p*=0.006), suggesting that this is a better potential reason for variation in camp stability.

*Table S1*: A list of all independent variables included in the ‘amount shared’ analysis.

| Variable | Level | Description |
| --- | --- | --- |
| Camp Stability | Camp | Measure of how much camp composition varied over multiple visits to the same camp, from 0 to 1, with 1 meaning no change in membership and 0 meaning complete change (see above). |
| Harvesting Rice | Camp | Whether members of camp were engaged in harvesting rice for non-Agta farmers. |
| Stored Rice | Individual | Whether the household had any rice stored or not |
| Money | Individual | Whether the household possessed any money (dichotomised to yes or no) |
| Camp Size | Camp | Number of families in camp |
| Sample relatedness to Ego | Individual | Average relatedness to ego (player) of individuals in the sample. A high value indicates increased average relatedness. |
| # of Primary Kin in Camp | Individual | Number of ego’s adult primary kin residing in camp |
| Sample Affine Depth to Ego | Individual | Average affine depth to ego (player) of sample (excluding consanguineal kin). Spouse = 1, Spouse’s primary kin or primary kin’s spouse = 2, spouses distant kin or other affines (up to 5 degrees of separation) = 3, not related = 4. A higher average value indicates less affinal relatedness. |
| Sex | Individual | Sex |
| Age | Individual | Age |
| # Dependent Offspring | Individual | Number of dependent offspring residing with parents (can also include grandchildren/adoptions) approx. aged <15 |
| Distance to Town | Camp | Distance to main town (kms) |
| Distance to Trade | Camp | Distance to nearest trade point (kms) |
| Church in Camp | Camp | Whether there was a Born Again church present in camp or not |
| Area | Camp | Whether camp was located coastal or inland |
| Height/Weight Ratio (BMI) | Individual | Measure of body size (weight (kg)/height(metres)^2^) |
| Sample Proximity to Ego | Individual | Average neighbourhood proximity to sample (0 = same house, 1 = next door, 2 = next door but 1, 3 = Further away). High average values indicate increasing distance from ego |
| Wealth | Individual | Amount of belongings (e.g., cooking pot, speargun, blanket, etc.) an individual has using transformed Z-scores. A total of 16 items were asked for each household, of which the 10 items the Agta rated as most important were used. Each of these 10 items was scored as ‘1’ if the amount was >1 standard deviation (SD) below the average amount, ‘2’ if between +/-1 SD, and ‘3’ if >1 SD above the average amount. These were averaged over all 10 items to produce a score between 1 (few items) and 3 (many items). |
| Education | Individual | 1 = No education/kindergarten, 2 = Primary education, 3 = High School |
| Primary Kin Score | Individual | Average amount kept for self in game by ego’s primary kin (relatedness coefficient=0.5; i.e., parents, children, or siblings) |
| Hunting Involvement | Individual | Of time spent observed during camp scans (4 per day, 3 hours apart, for a minimum of 6 days), the proportion of time spent engaged in subsistence activities for hunting |
| Fishing Involvement | Individual | Of time spent observed during camp scans (4 per day, 3 hours apart, for a minimum of 6 days), the proportion of time spent engaged in subsistence activities for fishing |
| Gathering Involvement | Individual | Of time spent observed during camp scans (4 per day, 3 hours apart, for a minimum of 6 days), the proportion of time spent engaged in subsistence activities for gathering wild plants |
| Hunting/Gathering Involvement | Individual | Of time spent observed during camp scans (4 per day, 3 hours apart, for a minimum of 6 days), the proportion of time spent engaged in subsistence activities for hunting, fishing, or gathering (combined) |
| Cash Labour Involvement | Individual | Of time spent observed during camp scans (4 per day, 3 hours apart, for a minimum of 6 days), the proportion of time spent engaged in subsistence activities for cash labour |
| Agriculture Involvement | Individual | Of time spent observed during camp scans (4 per day, 3 hours apart, for a minimum of 6 days), the proportion of time spent engaged in subsistence activities for agriculture |

*Table S2:* Sample sizes and summary statistics for each camp. An ‘A’ next to camp number indicates that this was one of the 11 camps used in the ‘amount shared’ analyses for which camp stability data were available.

| Camp | Sample Size (*n*) | Number of males | Mean % rice kept in Sharing Game (SD) | Mean % rice taken in Taking Game (SD) |
| --- | --- | --- | --- | --- |
| 54 (A) | 7 | 3 | 40.8 (9.9) | 40.8 (11.6) |
| 62 (A) | 9 | 5 | 47.2 (10.4) | 61.1 (22.4) |
| 64 (A) | 16 | 8 | 43.1 (29.4) | 44.6 (30.8) |
| 66 (A) | 26 | 14 | 61.5 (32.8) | 67.2 (26.8) |
| 67 (A) | 12 | 6 | 97.5 (6.2) | 94.4 (19.2) |
| 67.2 (A) | 18 | 8 | 78.3 (22) | 76.7 (26.5) |
| 74 (A) | 38 | 19 | 71.8 (26.4) | 61.6 (28.2) |
| 77 (A) | 10 | 4 | 93.3 (21.1) | 94.6 (17) |
| 78 (A) | 14 | 7 | 100 (0) | 96.7 (12.5) |
| 79 (A) | 23 | 11 | 69.6 (31.7) | 73.9 (32.2) |
| 84 (A) | 10 | 5 | 41 (17.3) | 42 (14.8) |
| 59 | 44 | 20 | 68.6 (24) | 67.9 (26.7) |
| M1 | 15 | 5 | 30.7 (17.5) | 56.9 (34.4) |
| M2 | 14 | 8 | 50.7 (29.2) | 47.6 (28.6) |
| M3.1 | 8 | 4 | 39.3 (27.3) | 35.6 (15.1) |
| M3.3 | 10 | 5 | 43 (19.9) | 75.4 (28.4) |
| M4 | 8 | 4 | 42.9 (20.2) | 62.5 (31.2) |
| M5 | 8 | 4 | 26.8 (20.8) | 44.5 (24.4) |
| Total (A) | 183 | 90 | 69.1 (30.1) | 68.7 (30) |
| Total (All) | 290 | 140 | 62.6 (30.5) | 65.4 (30) |

*Table S3*: Results of the univariate ‘amount shared’ analyses (*n*=183, camps=11) for both the Sharing Game and the Taking Game. Cells contain AIC values and whether the variable is significant compared to the null model. An “(M)” after the variable name signifies that this variable contained missing data, and AIC and significance values for these variables are averaged across each of the 5 imputed datasets. Cells in bold indicate variables with a *p*-value of <0.1, which are subsequently used in the model-averaging procedure. *p*-value codes: **˙** <0.1, * <0.05, ** <0.01, *** <0.001.

| Variable | Sharing Game | Taking Game |
| --- | --- | --- |
| Null | 1725 | 1734.5 |
| Camp Stability | **1711.9 ***** | **1718.9 ***** |
| Harvesting Rice | **1720.9 **** | **1731.3 *** |
| Distance to Town | **1711.1 ***** | **1721.9 ***** |
| Distance to Trade | 1724.9 | 1735 |
| Camp Size | 1726.8 | 1736.5 |
| Church in Camp | 1726.2 | 1735 |
| Area | 1726.2 | 1736.5 |
| Sex | 1725.6 | 1734.8 |
| Age | 1727 | 1736.1 |
| # Dependent Offspring | **1721.6 *** | **1726.7 **** |
| Height/Weight Ratio (M) | 1726.9 | 1736.4 |
| Sample relatedness to Ego | 1726.9 | 1735 |
| # of Primary Kin in Camp | **1724.1 ˙** | 1735.4 |
| Sample Affine Depth to Ego | **1721.5 *** | **1732.5 *** |
| Stored Rice (M) | **1723.6** **˙** | **1731.2 *** |
| Sample Proximity to Ego | **1723.8 ˙** | 1735.8 |
| Wealth (M) | 1726.9 | 1735.7 |
| Education (M) | 1726.2 | 1734.6 |
| Primary Kin Score (M) | 1726.1 | 1734.5 |
| Money (M) | 1727 | 1736.2 |
| Hunting Involvement (M) | 1726.9 | 1736.2 |
| Fishing Involvement (M) | 1725.9 | **1731.4 *** |
| Gathering Involvement (M) | 1726.9 | **1730.1 *** |
| Hunting/Gathering Involvement (M) | 1725.8 | 1736.3 |
| Cash Labour Involvement (M) | **1720.5 *** | 1736.1 |
| Agriculture Involvement (M) | 1726.1 | 1736.2 |

*Table S4*: Colinearity in SG and TG for the variables entered into the model-averaging ‘amount shared’ analysis. Variance inflation factors (VIFs) for each variable are pooled across each of the five imputed datasets. A VIF>3 is indicative of high colinearity, which may bias parameter estimates [21]. None of the VIFs reported here are above 2, indicating that colinearity between predictor variables is not biasing the results.

| Variable | Sharing Game VIF | Taking Game VIF |
| --- | --- | --- |
| Camp Stability | 1.48 | 1.13 |
| # Dependent Offspring | 1.1 | 1.11 |
| Harvesting Rice | 1.56 | 1.28 |
| Stored Rice | 1.07 | 1.07 |
| # Primary Kin in Camp | 1.18 | NA |
| Affinal Closeness | 1.46 | 1.09 |
| Average Proximity to Sample | 1.97 | NA |
| Cash Labour Involvement | 1.23 | NA |
| Fishing Involvement | NA | 1.33 |
| Gathering Involvement | NA | 1.25 |

*Table S5:* Comparison of QIC values to find the best-fitting model for the SG (*n*=290, dyads=1,312). Note that all models, including the control, contain the amount given by ego to control for differences in amount given between individuals. The best-fitting model (lowest QIC value) is highlighted in bold.

| Model | QIC Value |
| --- | --- |
| Control | 1126.52 |
| Reciprocity | 1115.96 |
| Kinship | 1102.29 |
| Proximity | 1126.62 |
| Reciprocity and Kinship | **1098.58** |
| Reciprocity and Proximity | 1117.04 |
| Kinship and Proximity | 1104.38 |
| Reciprocity, Kinship, and Proximity | 1100.77 |

*Table S6:* Comparison of QIC values to find the best-fitting model for the TG (*n*=290, dyads=1,312). Note that all models, including the control, contain the amount taken by ego to control for differences in amount taken between individuals. The best-fitting model (lowest QIC value) is highlighted in bold.

| Model | QIC Value |
| --- | --- |
| Control | 1107.08 |
| Reciprocity | 1109.45 |
| Kinship | 1113.01 |
| Proximity | 1106.71 |
| Resource Quantity | 912.94 |
| Reciprocity and Kinship | 1115.18 |
| Reciprocity and Proximity | 1109.12 |
| Reciprocity and Resource Quantity | 914.69 |
| Kinship and Proximity | 1115.18 |
| Kinship and Resource Quantity | 915.93 |
| Proximity and Resource Quantity | **911.52** |
| Reciprocity, Kinship, and Proximity | 1117.21 |
| Reciprocity, Kinship, and Resource Quantity | 917.46 |
| Reciprocity, Proximity, and Resource Quantity | 913.27 |
| Kinship, Proximity, and Resource Quantity | 917.06 |
| Reciprocity, Kinship, Proximity, and Resource Quantity | 918.63 |

*Table S7*: Dates of camp censuses and length of time between visits when constructing the ‘camp stability’ metric. Stability scores vary between ‘0’ (complete change in camp composition between visits) and ‘1’ (no change in camp composition between visits). Note that there are no systematic biases in either the census dates or the length of time between visits for stable and unstable camps. Note also that all visits to camps were made in the dry season (from February to September). Seasonality or sampling biases are therefore unlikely to influence variation in camp stability.

| Camp | Stability Score | Dates of Census | Length of Time Between Visits |
| --- | --- | --- | --- |
| 54 | 0.6 | 05/2013, 03/2014, & 09/2014 | 10 months & 6 months |
| 62 | 0.525 | 06/2013, 04/2014, & 09/2014 | 10 months & 5 months |
| 64 | 0.774 | 05/2013, 04/2014, & 09/2014 | 11 months & 5 months |
| 66 | 0.732 | 04/2013, 04/2014, 06/2014, & 09/2014 | 12 months, 2 months, & 3 months |
| 67 | 0.21 | 04/2013 & 07/2014 | 15 months |
| 67.2 | 0.509 | 04/2013 & 07/2014 | 15 months |
| 74 | 0.612 | 05/2013, 05/2014, & 08/2014 | 12 months & 3 months |
| 77 | 0.327 | 06/2014 & 08/2014 | 2 months |
| 78 | 0.122 | 06/2014 & 08/2014 | 2 months |
| 79 | 0.384 | 06/2014 & 08/2014 | 2 months |
| 84 | 0.789 | 06/2013, 03/2014, & 08/2014 | 9 months & 5 months |

**A**

**F**

**B**

**C**

**D**

**E**

**G**

**A**

**F**

**B**

**C**

**D**

**E**

**G**

*Figure S1*: Schematic visualisation of the Sharing Game (upper) and Taking Game (lower) prior to play in a camp where *n* = 8 (i.e., ego plus 7 camp-mates). Grey circles represent rice tokens and arrows denote direction in which the tokens are to be moved.


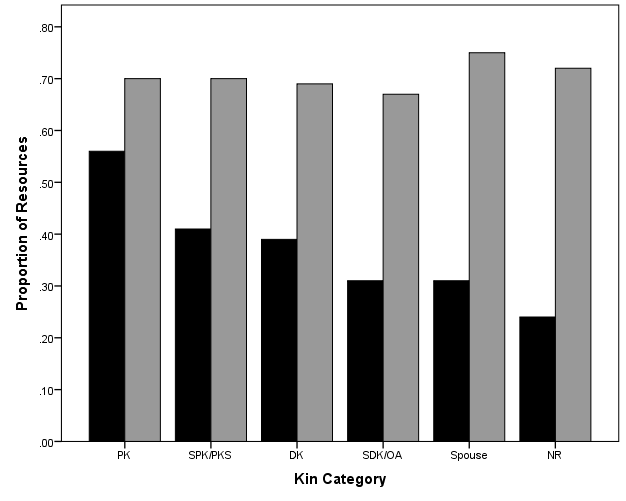


*Figure S2*: Average proportion of resources given to (black; Sharing Game) or taken from (grey; Taking Game) as a function of kin category (PK = primary kin; SPK/PKS = spouse’s primary kin/primary kin’s spouse; DK = distant kin; SDK/OA = spouse’s distant kin/other affines; NR = No relation: *n*=290, dyads=2,752).

References for Supplementary Material

1. Bellwood, P. 1999 Archaeology of South-East Asian hunters and gatherers. In *The Cambridge Encyclopedia of Hunters and Gatherers* (eds R. B. Lee & R. Daly), pp. 284–288. Cambridge, MA: Cambridge University Press.

2. Apicella, C. L., Marlowe, F. W., Fowler, J. H. & Christakis, N. A. 2012 Social networks and cooperation in hunter-gatherers. *Nature* **481**, 497–501. (doi:10.1038/nature10736)

3. Chaudhary, N., Salali, G. D., Thompson, J., Dyble, M., Page, A., Smith, D., Mace, R. & Migliano, A. B. 2015 Polygyny without wealth : popularity in gift games predicts polygyny in BaYaka Pygmies Subject Category : *R. Soc. Open Sci.* **2**, 150054.

4. Thomas, M. G., Næss, M. W., Bårdsen, B.-J. & Mace, R. 2015 Saami reindeer herders cooperate with social group members and genetic kin. *Behav. Ecol.* **26**, 1495–1501. (doi:10.1093/beheco/arv106)

5. Kollock, P. 1998 Social Dilemmas: The Anatomy of Cooperation. *Annu. Rev. Sociol.* **24**, 183–214. (doi:10.1146/annurev.soc.24.1.183)

6. Camerer, C. F. 2003 *Behavioral Game Theory: Experiments in Strategic Interaction*. Princeton University Press.

7. Rucas, S. L., Gurven, M., Kaplan, H. & Winking, J. 2010 The social strategy game. *Hum. Nat.* **21**, 1–18. (doi:10.1007/s12110-010-9079-z)

8. Marlowe, F. W. 2005 Hunter-gatherers and human evolution. *Evol. Anthropol.* **14**, 54–67. (doi:10.1002/evan.20046)

9. Minter, T. 2010 The Agta of the Northern Sierra Madre: Livelihood Strategies and Resilience among Philippine Hunter-Gatherers.

10. Team, R. D. C. 2015 R: A language and environment for statistical computing.

11. Bates, D., Maechler, M., Ben, B. & Walker, S. 2015 Package ‘lme4’.

12. Kreft, I. & de Leeuw, J. 1998 *Introducing Multilevel Modelling*. Sage.

13. Barton, K. 2015 MuMIn: Multimodel Inference.

14. Grueber, C. E., Nakagawa, S., Laws, R. J. & Jamieson, I. G. 2011 Multimodel inference in ecology and evolution: Challenges and solutions. *J. Evol. Biol.* **24**, 699–711. (doi:10.1111/j.1420-9101.2010.02210.x)

15. Burnham, K. P. & Anderson, D. R. 2002 *Model Selection and Multimodel Inference: A Practical Information Theoretic Approach*. Springer Science and Business Media.

16. Honaker, J., King, G. & Blackwell, M. 2011 AMELIA II : A Program for Missing Data. *J. Stat. Softw.* **45**, 1–54. (doi:10.1.1.149.9611)

17. Rubin, D. B. 2004 *Multiple Imputation for Non-Response in Surveys*. John Wiley & Sons.

18. Nakagawa, S. & Freckleton, R. P. 2011 Model averaging, missing data and multiple imputation: A case study for behavioural ecology. *Behav. Ecol. Sociobiol.* **65**, 103–116. (doi:10.1007/s00265-010-1044-7)

19. Dyble, M., Salali, G. D., Chaudhary, N., Page, A., Smith, D., Thompson, J., Vinicius, L., Mace, R. & Migliano, A. B. 2015 Sex equality can explain the unique social structure of hunter-gatherer bands. *Science* **348**, 796–798.

20. Dyson-Hudson, R. & Smith, E. A. 1978 Human Territoriality: An Ecological Reassessment. *Am. Anthropol.* **80**, 21–41.

21. Zuur, A. F., Ieno, E. N. & Elphick, C. S. 2010 A protocol for data exploration to avoid common statistical problems. *Methods Ecol. Evol.* **1**, 3–14. (doi:10.1111/j.2041-210X.2009.00001.x)
